# Implant failure and history of failed endodontic treatment: A retrospective case-control study

**DOI:** 10.4317/jced.54277

**Published:** 2017-11-01

**Authors:** Georgios S. Chatzopoulos, Larry F. Wolff

**Affiliations:** 1 DDS, Advanced Education Program in Periodontology, Department of Developmental and Surgical Sciences, School of Dentistry, University of Minnesota, 515 Delaware St. SE, Minneapolis, MN 55455; 2 MS, PhD, DDS, Professor, Division of Periodontology, Department of Developmental and Surgical Sciences, School of Dentistry, University of Minnesota, 515 Delaware St. SE, Minneapolis, MN 55455

## Abstract

**Background:**

Residual bacterial biofilm and/or bacteria in planktonic form may be survived in the bone following an extraction of an infected tooth that was endodontically treated unsuccessfully Failed endodontic treatment may be associated with failure of implants to osseointegrate in the same sites. Therefore, the aim of this retrospective case-control study is to examine the risk of implant failure in previous failed endodontic sites.

**Material and Methods:**

This retrospective case-control study is based on 94 dental records of implants placed at the University of Minnesota School of Dentistry. Dental records of patients who received an implant in sites with previously failed endodontic therapy in the dental school were identified from the electronic database, while control subjects were obtained from the same pool of patients with the requirement to have received an implant in a site that was not endodontically treated.

**Results:**

The mean age of the population was 62.89±14.17 years with 57.4% of the sample being females and 42.6% of them being males. In regards to the socio-economic status and dental insurance, 84.0% of this population was classified as low socio-economic status and 68.1% had dental insurance. Tobacco use was self-reported by 9.6% and hypercholesterolemia was the most prevalent systemic medical condition. Dental implant failure was identified in two of the included records (2.1%), both of which were placed in sites with a history of failed endodontic treatment.

**Conclusions:**

Within the limitations of this retrospective case-control study, further investigation with a larger population group into implant failure of sites that previously had unsuccessful endodontic treatment would be warranted. Implant failure may be associated with a history of failed endodontic treatment.

** Key words:**Implantology, endodontics, osseointegration, treatment outcome, case-control study.

## Introduction

Dental implants often represent the best treatment modality to replace a single or multiple missing teeth due to their predictable outcomes and effectiveness (1,2). Implants provide not only function but also improved esthetics and frequently reduced psychological trauma compared to conventional restorative treatment options (3,4). Although implants have shown high success rates, failures during the initial healing periods and during loading and maintenance have been reported (5). Evidence from long-term studies presents an incidence rate of peri-implantitis that ranges from 11.2% to 47.1% of implants and a number of those will eventual fail, if not treated (6). Implant failures have been associated with patients’ risk factors such as a patient’s systemic condition, pathogenic bacteria, implant macro- and micro-design, smoking, bone quality, number of implants placed as well as their distribution (5,7,8). The identification by the dental professional of key factors related to the etiology and risk of peri-implantitis and implant failure may minimize the risk of failure and improve the therapeutic approach in placing implants.

Failure of dental implants are characterized by clinical and radiographic signs that include pain, mobility, peri-implant radiolucency and excessive loss of alveolar bone (9). Early failure may occur during the healing period and has been associated with poor osseointegration, while late failure may occur after the implant is osseointegrated and may be the result of inadequate bone preservation (5). Radiolucency around an implant with lack of bone-to-implant contact and mobility are evident in failed implants, whereas failing implants show continuous slow marginal bone loss with lack of implant mobility (5). Implant failure has been also associated with apical peri-implantitis which is a result of previous failed endodontic treatment and extraction of a natural tooth due to presence of persistent periapical pathology (10). This finding confirm previous reports that bacterial colonization may lead to implant failures (11,12). After extraction of the failed endodontically treated tooth, a residual bacterial infection may remain and not eliminated before the implant is placed.

Non-surgical endodontic treatment is a predictable treatment option that exhibits success rates between 86% and 98% (13,14). In case of teeth with periapical pathology, a success rate of 75% was reported by Ng and colleagues, when complete absence of periapical radiolucency was considered, while the rate was increased to 85% when reduction in the size of the periapical radiolucency was adopted in the criteria for the analysis (15). Ten-year endodontic complications have been reported in approximately 4% of endodontically treated teeth and include symptoms, swelling and need for re-treatment (16). Endodontically treated teeth may fail due to a variety of reasons that include persistent or reintroduced intraradicular microorganisms, extraradicular infection, foreign body reaction and true cysts. Failure may occur due to iatrogenic endodontic procedural errors, complications of instrumentation or anatomical difficulties (17). Treatment options after an initial failed root canal treatment include non-surgical re-treatment, endodontic surgery, tooth replantation, transplantation or extraction and replacement with either a single implant or with a fixed prosthesis (18). Extraction and replacement with a dental implant may occur either immediately (immediate implant placement) or in a two-stage approach that will allow the site to heal before the implant is placed.

Failed endodontic treatment may be associated with failure of implants to osseointegrate in the same sites. Therefore, the aim of this retrospective case-control study is to examine the risk of implant failure in previous failed endodontic sites.

## Material and Methods

-Subject population

This retrospective case-control study is based on data obtained from the electronic records at the University of Minnesota School of Dentistry for treatment provided by dental students, residents and faculty to patients attending the dental clinics between 2010 and 2016. The Institutional Review Board of the University of Minnesota School of Dentistry approved the present study as a medical record chart review. Dental records of patients who received an implant in sites with previously failed endodontic therapy in the dental school were identified from the electronic database of the School of Dentistry. Patients were examined for potential inclusion in the study only if they were 18 years of age at the time of implant therapy and complete information about the demographic and implant characteristics, insurance status and medical history was available. Control subjects were obtained from the same pool of patients with the requirement to have received an implant in a site that was not endodontically treated.

-Data collection

Patient’s information was recorded including chart number, age at the time of the treatment, gender, presence/absence of dental insurance, medical history, tobacco use and ZIP code. The examined systemic medical conditions consisted of self-reported hypertension, heart attack, hypercholesterolemia, asthma, diabetes mellitus, thyroid disorder, kidney disorder, arthritis, artificial joint, osteoporosis, depression, anxiety, cancer and cancer treatment. Implant characteristics were also obtained including implant diameter and length, arch (maxilla, mandible), region (anterior, posterior), implant system (Zimmer, Astra, Nobel, 3i, Straumann) as well as the time between implant placement and 2nd stage surgery. Patient ZIP codes were used to evaluate socio-economic status based on the 2010-2014 American Community Survey 5-year estimates of the U.S. Census Bureau. The patients were classified with a low and high socio-economic status based on the mean income of the total population.

-Type of treatment

The ADA codes were utilized to identify patients that had received endodontic and implant treatment in the same site. Patients were considered to have a history of endodontic treatment based on the following ADA codes: D3310 (endodontic therapy, anterior, primary tooth), D3320 (endodontic therapy, bicuspid tooth), D3330 (endodontic therapy, molar 3 canal), D3330B (molar 4 canal), D3346 (retreatment of previous root canal therapy-anterior), D3347 (retreatment of previous root canal therapy-bicuspid), D3348 (retreatment of previous root canal therapy-molar), D3351 (apexification/Recalcification-initial visit), D3355 (pulpal regeneration- initial visit), D3410 (apicoectomy-anterior), D3421 (apicoectomy-bicuspid), D3425 (apicoectomymolar), D3450 (root amputation), D3470 (intentional reimplantation), D7270 (tooth reimplantation/stabilize). Implant treatment was identified based on the D6010 code (surgical placement, endosteal implant). All included implants were surgically placed by residents or faculty in the Division of Periodontology, Oral and Maxillofacial surgery, Prosthodontics and Endodontics at the University of Minnesota School of Dentistry. On the other hand, endodontic treatments were performed by dental students, graduate students in the Division of Endodontics or faculty at the University of Minnesota School of Dentistry faculty practice clinic. All patients that had received implant treatment following a failed endodontic treatment (n=47) were included in the analysis, while 47 patients with an implant treatment in a site without history of endodontic therapy were randomly selected to serve as control.

-Statistical analysis

The data from the included dental charts were collected and imported in a computer database and analyzed utilizing a statistical program. The primary endpoint of the study was implant failure in patients with and without a history of endodontic treatment in the site of implant placement. Descriptive statistics including frequencies, means and standard deviations were calculated for the demographic characteristics. Treatment outcome and history of endodontic treatment was assessed by chi-square test. Chi-square test was also utilized to examine the differences between test (implants placed in sites with failed endodontic tre-atment) and control (implants placed in sites without a history of endodontic treatment) groups. All tests of significance were evaluated at the 0.05 error level with a statistical software program (SPSS v.24.0, IBM, Armonk, NY, USA).

## Results

A total of 47 dental records of implants placed in sites with previous failed endodontic treatment were identified in the electronic database of the University of Minnesota and included in the test group. Records of implants placed in sites without a history of endodontic treatment were initially screened for eligibility after considering the inclusion and exclusion criteria of the present study and 47 randomly selected records were included in the control group. Hence, a total of 94 records of dental implants were ultimately included in the final analysis to examine whether a history of failed endodontic treatment is associated with implant failure.

The characteristics of the study population are shown in  Table 1,  Table 1 continue. The mean age of the included population was 62.89±14.17 years with 57.4% of the sample being females and 42.6% of them being males. In regards to the socio-economic status, 84.0% were classified as low, while 16.0% as high status. With respect to insurance, 68.1% of the included sample had dental insurance, whereas 31.9% did not. Tobacco use was self-reported by 9.6% and hypercholesterolemia was the most prevalent among the other examined systemic medical conditions in the sample. Approximately one-third of the sample reported medical history significant for hypertension (30.9%), hypercholesterolemia (35.1%) and arthritis (28.7%). Diabetes mellitus was reported by 9.6% of the examined population and osteoporosis by 7.4%.

**Table 1:**
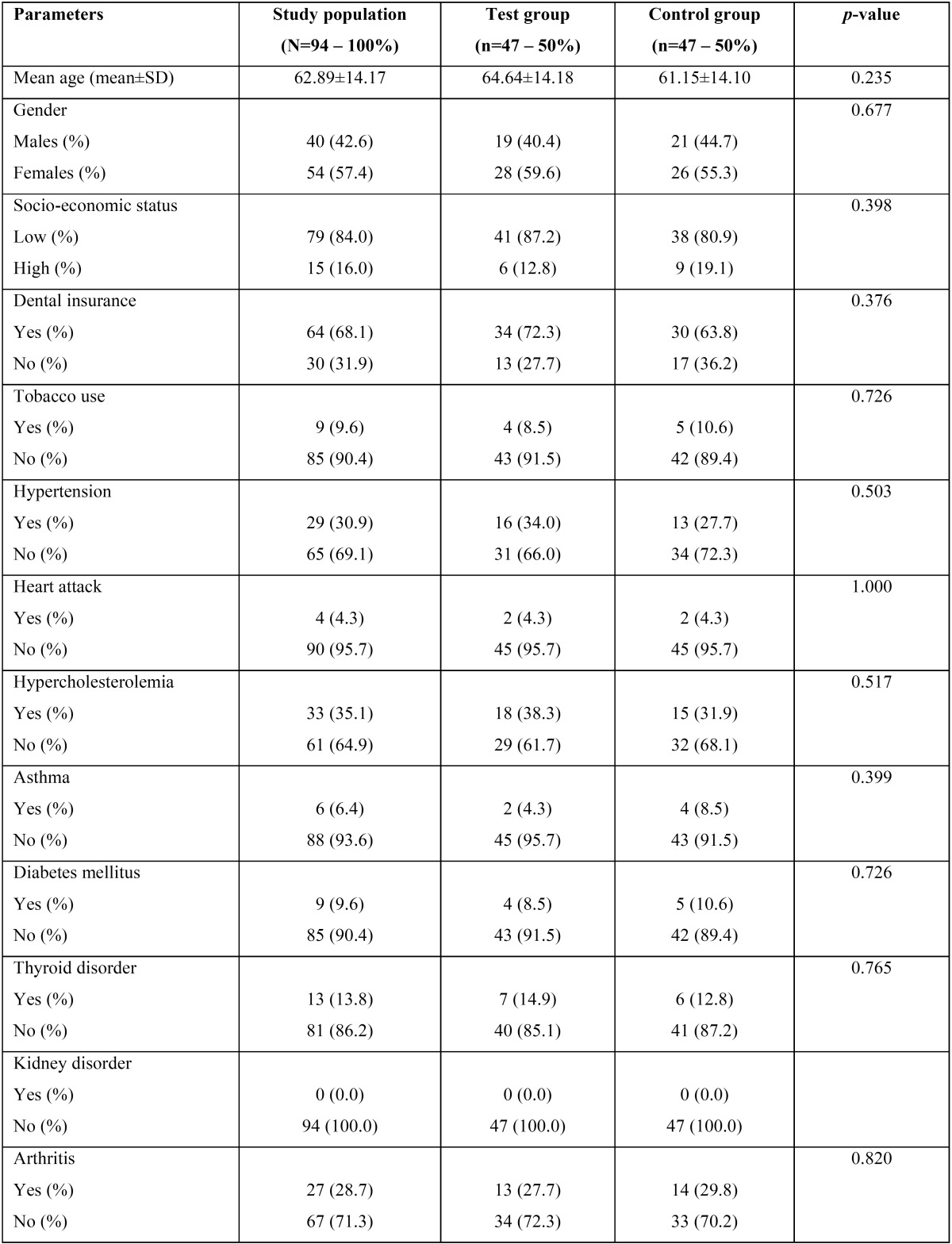
Characteristics (demographic and systemic conditions) of the study population.

**Table 1 continue:**
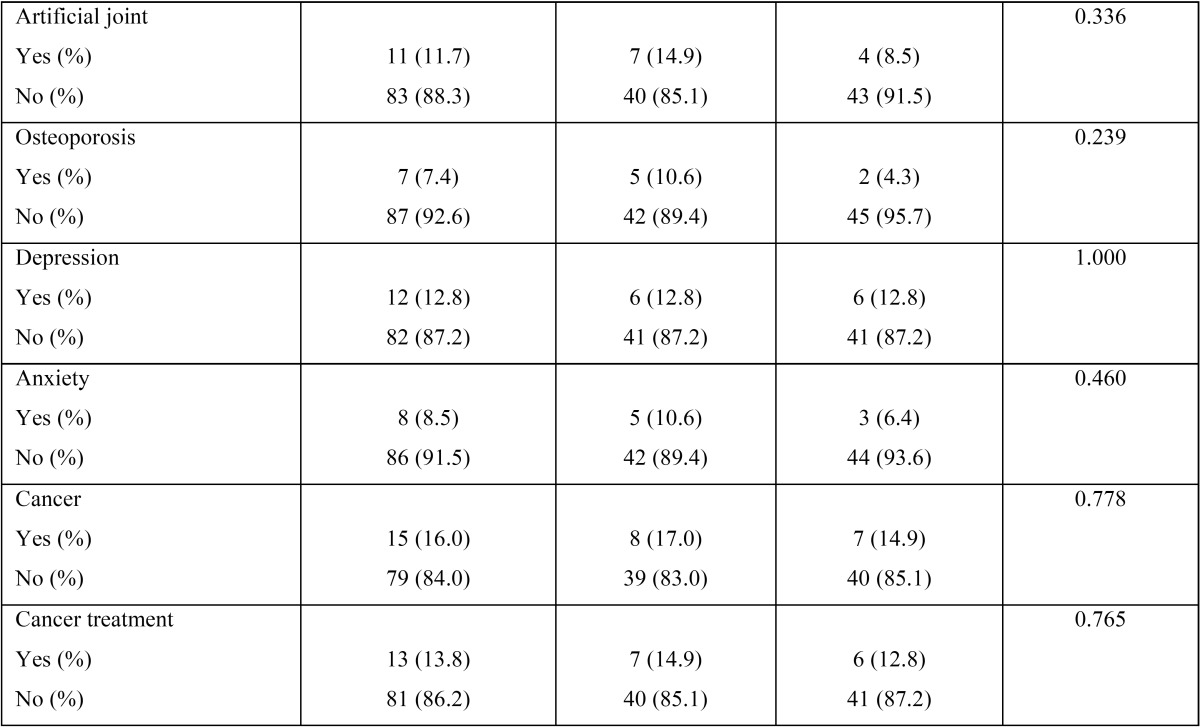
Characteristics (demographic and systemic conditions) of the study population.

The characteristics of the examined implants are shown in  Table 2. With respect to the location of the implants, 50% of them were in the maxilla and 72.3% in a posterior region. Of the 94 included implants, 84% was associated with a >10 mm length and 68.1% with a >4 mm diameter. The most commonly placed implant system was Zimmer Biomet ScrewVent TSVT (57.4%). The time between implant placement and 2nd stage implant surgery was 2.53±2.61 months, while 45.75% of the implants were non-submerged. Dental implant failure was identified in two of the included records (2.1%).

**Table 2:**
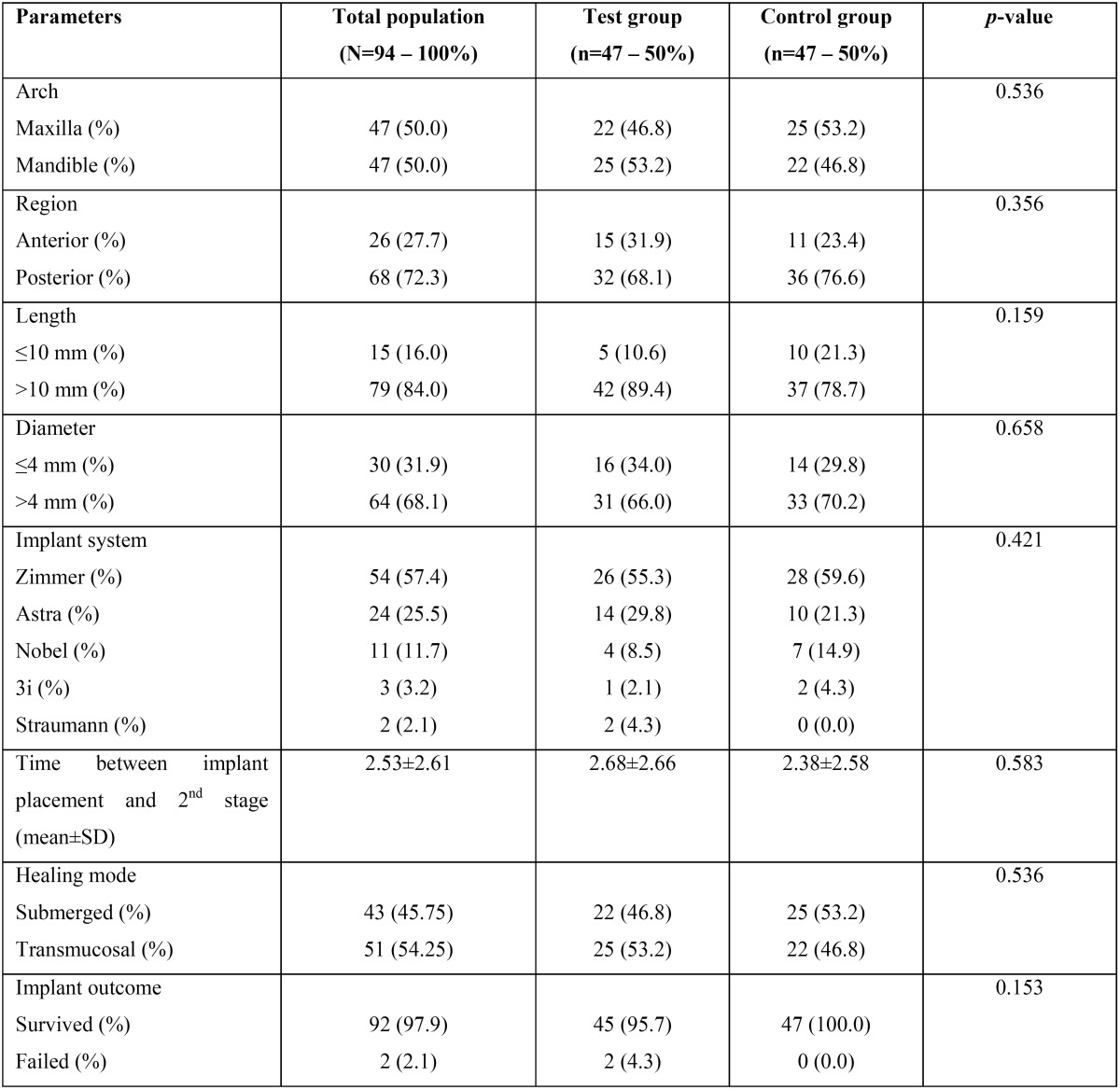
Characteristics of the examined dental implants.

Both failed implants were placed in sites with a history of failed endodontic treatment, but due to the small sample size, this difference did not reach the level of significance (*p*=0.153). Failed implants followed a non-submerged healing mode, were >10 mm in length and were placed in the maxilla. Both individuals, that experienced implant failure, followed a history of failed endodontic treatment and were males with a mean age of 56.0±32.53 years.

## Discussion

In this study, a total of 94 records of dental implants placed at the University of Minnesota School of Dentistry were included to assess the potential association between history of endodontic treatment and implant therapy outcome. The authors hypothesized that sites with a history of failed root canal treatment will affect negatively implant survival. The electronic chart review of implants from the University of Minnesota School of Dentistry revealed that 47 patients received an implant in a site with endodontic treatment failure and to test our hypothesis another 47 patients who had implant placement without any complications were randomly selected to eliminate any potential risk of bias. The results of the current study showed that two out of the 94 included implants failed. Both of these implants were placed in sites with a history of endodontic failure. However, no significant difference was detected between the two groups as a result of the small number of examined records.

Residual bacterial biofilm and/or bacteria in planktonic form may be survived in the bone following an extraction of an infected tooth that was endodontically treated unsuccessfully (12). Failed root canal treated teeth are commonly associated with facultative anaerobic gram-positive bacterial species which is a result of a change in the bacterial population and microbial metabolic characteristics (19). A typical treatment approach in everyday clinical practice when a failed endodontic tooth is extracted, is to debride thoroughly the socket and either fill it with bone graft or immediately place a dental implant. Pathogenic bacteria that have not been completely removed may survive and reach a vegetative state in the alveolar cancellous bone, where the bacteria may then propagate after the site is drilled during implant placement (11,12). Residual bacteria may then colonize the implant surface which eventually will lead to implant failure (11,12).

Limited information is available in the dental literature in regards to the effect of pulp pathology on implant treatment outcome. Cases with peri-implantitis lesions were reported by Romanos *et al.* in areas with previous endodontic treatment and these investigators concluded that “areas with endodontically compromised teeth might interfere with implant success” and this risk is present regardless of the healing time allowed following the extraction of the natural tooth (20). In an observational retrospective study, although implants can be placed successfully in sites with no history of endodontic treatment with a 97.6% success rate, 50.41% of the implants placed in sites with and/or adjacent to previous endodontic failures demonstrated signs of peri-implantitis. Implants placed in the maxilla demonstrated a higher prevalence of peri-implantitis than in the mandible possibly due to the quality of cortical bone (11,21). In agreement with this finding, both of the included implant failures in the present study were in the maxilla.

In the case of a failing endodontic therapy, treatment options include monitoring, re-treatment or extraction (22). Implant failures in dentistry have been associated with loss of osseointegration, poor treatment planning, poor surgical treatment which is attributed by the implant positioning, soft tissue defects and biomechanical complications (23). Implant failure leads to an additional cost for the patient, the need of further treatment which causes frustration for the patient as well as the clinician. Patient selection and proper treatment planning are of paramount importance to achieve long-term implant success and survival.

## Conclusions

Within the limitations of this retrospective case-control study, there might be an association between failed endodontic treatment and dental implant failure. Presence of residual bacteria in the surrounding bone adjacent to implants as a result of a history of failed endodontic treatment may induce implant failure. Further prospective large-scale studies should be performed to examine this hypothesis.
